# Authors' reply

**Published:** 2010

**Authors:** Vinay B Agrawal

**Affiliations:** Clear Vision Eye Center, 1A, Ashoka, Santacruz (W), Mumbai - 400 054, Maharashtra, India

Dear Editor,

I thank Parthasarathy[1] for the specific interest in our work[2] on corneal collagen cross-linking with riboflavin. This study was a part of a larger study on various aspects of keratoconus at our center. Thus, vernal keratoconjunctivitis was not specifically mentioned in this article. In another original paper submitted for publication, we have reported that 24.4% of patients with keratoconus had symptomatic ocular allergies.

The seven eyes did not qualify for inclusion in the stable or improved category as the criteria mentioned was ±0.5 DS. If the criterion was changed to ±1.5 D, all eyes remained stable in our study. Thus, no patient lost best corrected visual acuity (BCVA). Change in refraction after treatment is a common finding in our patients (unpublished data). Of the seven eyes in question, only two had co-existing allergic eye disease.

Postoperative ultrasonic pachymetry has been performed for each patient at each follow-up visit. The change pattern was not significant, as seen from the Table 1 of the published article.[1]

About the values showing a large standard deviation, it is due to the changes in keratometry in various patients. If we look individually, we have had patients showing responses varying from up to a 7 D change in astigmatism to no change. Thus the large standard deviations in the statistical analysis. The BCVA did not drop in our series, as mentioned earlier.

The issue of corneal wavefront evaluations is the subject of another ongoing study at our center.
